# Analysis of patient data from laboratories during the Ebola virus disease outbreak in Liberia, April 2014 to March 2015

**DOI:** 10.1371/journal.pntd.0005804

**Published:** 2017-07-21

**Authors:** Yuki Furuse, Mosoka Fallah, Hitoshi Oshitani, Ling Kituyi, Nuha Mahmoud, Emmanuel Musa, Alex Gasasira, Tolbert Nyenswah, Bernice Dahn, Luke Bawo

**Affiliations:** 1 Frontier Research Institute for Interdisciplinary Sciences, Tohoku University, Sendai, Japan; 2 Department of Virology, Tohoku University Graduate School of Medicine, Sendai, Japan; 3 Ministry of Health and Social Welfare, Monrovia, Liberia; 4 United Nations Office at Nairobi, Nairobi, Kenya; 5 World Health Organization, Monrovia, Liberia; Institute for Disease Modeling, UNITED STATES

## Abstract

An outbreak of Ebola virus disease (EVD) in Liberia began in March 2014 and ended in January 2016. Epidemiological information on the EVD cases was collected and managed nationally; however, collection and management of the data were challenging at the time because surveillance and reporting systems malfunctioned during the outbreak. EVD diagnostic laboratories, however, were able to register basic demographic and clinical information of patients more systematically. Here we present data on 16,370 laboratory samples that were tested between April 4, 2014 and March 29, 2015. A total of 10,536 traceable individuals were identified, of whom 3,897 were confirmed cases (positive for Ebola virus RNA). There were significant differences in sex, age, and place of residence between confirmed and suspected cases that tested negative for Ebola virus RNA. Age (young children and the elderly) and place of residence (rural areas) were the risk factors for death due to the disease. The case fatality rate of confirmed cases decreased from 80% to 63% during the study period. These findings may help support future investigations and lead to a fuller understanding of the outbreak in Liberia.

## Author summary

Liberia experienced a large Ebola outbreak from March 2014 to January 2016. Collection and management of the data on the outbreak were challenging because surveillance and reporting systems malfunctioned at the time. Diagnostic laboratories, however, were able to register important information independently and more systematically. We obtained data on 16,370 laboratory samples during the outbreak. Analyses of the data revealed that there were differences in sex, age, and place of residence between Ebola cases and non-Ebola suspected cases. Young children, the elderly, and patients living in rural areas were more likely to die because of the disease. Our analysis also found a decreasing trend in the case fatality rate of Ebola during the outbreak. These findings may help support future investigations and lead to a fuller understanding of the outbreak in Liberia.

## Introduction

Ebola virus (EBOV) causes a severe infection in humans, Ebola virus disease (EVD), causing various symptoms such as fever, hemorrhage, myalgia, and diarrhea [1,2]. Although some EVD-specific treatments, such as an antibody cocktail [3] and a viral polymerase inhibitor [4], are being developed, symptomatic and supportive care is presently the main form of treatment [2]. The case fatality rate (CFR) of the disease ranges from 24% to 89% [2,5–8].

Bats are considered the natural hosts of the virus [9], although this is controversial [10,11]. People can become infected through contact with infected wildlife, such as bats or monkeys [11]. Human-to-human transmission of the virus is also possible through contact with body fluids, such as blood and watery stools of patients [11]. In March 2014, an EVD outbreak was first reported in Guinea [12] and the virus then spread to neighboring countries in West Africa [8,13]. According to the World Health Organization (WHO), by March 30, 2016, the devastating outbreak had affected more than 28,000 individuals, resulting in over 11,000 deaths in 10 countries [14]. Liberia was one of the hardest-hit countries during the outbreak with 10,675 cases (total number of suspected, probable, and confirmed cases), among which 4,809 were fatal [14,15]. The outbreak in Liberia began in March 2014; human-to-human transmission linked to the most recent cluster of cases in Liberia was declared to have ended on January 14, 2016 [14].

Epidemiological information on suspected, probable, and confirmed EVD cases was collected and managed nationally in Liberia using Microsoft Excel (Microsoft Corp., Redmond, Washington, US), the Epi-Info Viral Hemorrhagic Fever Application [16], and the DHIS 2 (DHIS2, Oslo, Norway). However, surveillance and reporting systems malfunctioned during the outbreak [17], resulting in inadequate data management including missing and duplicate data.

During the outbreak, sick persons were reported from communities via telephone calls to Liberia’s Ebola hotline, and case investigation teams visited and interviewed patients to ascertain whether they were suspected or probable EVD cases. All patients who visited health facilities in Liberia were also triaged to ascertain whether they were suspected or probable EVD cases. When patients met the definition of suspected or probable cases, they were transferred to Ebola Treatment Units (ETUs) [15,18,19]. There, they were interviewed again to check their status as suspected or probable cases. Blood and oral swab samples from suspected and probable cases were sent to EVD diagnostic laboratories within the country for the detection of EBOV RNA by nucleic acid tests (NATs). Samples from dead bodies were also tested, as per a national policy which stated that all dead bodies should be swabbed and tested for EBOV RNA regardless of the cause of death, during the outbreak [15].

Using data from the diagnostic laboratories, we could distinguish between patients who tested positive for EBOV RNA (confirmed cases) and those who tested negative for EBOV RNA (termed EBOV RNA-negative suspected cases because the data could not differentiate between suspected and probable cases). In addition to results of NATs, the laboratories recorded basic demographic and clinical information of patients systematically, independent of the epidemiological information collected and managed nationally. Data from the laboratories was likely to be primary and thus more accurate. Here, we present an analysis of data from the EVD diagnostic laboratories on samples that were tested between April 4, 2014 and March 29, 2015.

## Methods

### Ethics statement

This investigation was performed as a part of the Ebola public health response in Liberia. It was not considered to be a research on human subjects, as per the US federal human subjects’ protection regulations. Because data were not collected for research purposes, but for public health response, raw data were not anonymized. Serial samples from the same individual were identified using the raw data. Thereafter, line listing of individuals was anonymized and used for analyses in this study.

### Outbreak case definition in Liberia

As described in [18], a suspected case of EVD was defined as a person with an illness characterized by a history of acute fever and three or more symptoms (among headache, nausea, vomiting, diarrhea, intense fatigue, abdominal pain, general muscular or joint pain, difficulty swallowing, difficulty breathing, and hiccups) or by fever with acute clinical symptoms or signs of hemorrhage. A probable case of EVD was defined as a person with an illness meeting the suspected case definition or with a fever following contact with a probable or confirmed case of EVD in the past 21 days. Probable cases also included dead persons with such a history or any unexplained cause of death. In our study, data from the laboratories did not enable us to distinguish between suspected and probable cases. A confirmed case of EVD was defined as a suspected or probable case confirmed by laboratory testing (detection of EBOV RNA).

A patient who had no positive NAT results for EBOV RNA was classed as an EBOV RNA-negative suspected case. All dead bodies were tested for EBOV RNA during the outbreak; therefore, dead bodies that tested negative for EBOV RNA may not have shown any symptoms to suspect EVD. Their cause of death could have been cardiac infarct, stroke, or even a traffic accident. Therefore, such cases were not regarded as EBOV RNA-negative suspected cases, but as EBOV RNA-negative cases (dead bodies). Patients who died after testing positive for EBOV RNA and dead bodies testing positive for EBOV RNA were classed as confirmed fatal cases. Patients who tested positive and then tested negative were considered confirmed survival cases.

### Data from laboratories

A total of 10 laboratories, operated by national and international partners, performed NATs to detect EBOV RNA in samples from suspected and probable cases and all dead bodies during the outbreak in Liberia (S1 Table). Two of the laboratories were in the capital area (Montserrado County), where a quarter of the national population resides [20], and eight were in separate rural areas, each receiving samples from their served area [15]. The population served by each laboratory could not be determined, because the areas changed during the course of the outbreak. Each laboratory sent data every day, including sample ID, patient ID, name, age, sex, status of the patient (dead or alive) at the time of sample collection, test results, sample type, date of test, and date of symptom onset, to the Ministry of Health and Social Welfare. These data were then integrated at the central level. Serial samples from the same patient were identified through a review of these data.

### Case fatality rate (CFR)

Because some cases lacked information on date of symptom onset, we calculated trimonthly CFRs based on the date of initial testing.

### Statistical analysis

The chi-square test was used to detect differences in the prevalence of demographic factors between confirmed and EBOV RNA-negative suspected cases as well as between confirmed fatal and confirmed survival cases. The Mann–Whitney U-test was used to compare medians of the time period from symptom onset to initial testing between confirmed and EBOV RNA-negative suspected cases. The Mantel–Haenszel test for trend was performed for trimonthly CFR among confirmed cases. Binomial logistic regression analysis was performed to calculate risk of fatality due to the time period from symptom onset to initial testing. Missing data were excluded for the analyses. All tests were performed using SPSS version 24 (IBM Corp., Armonk, New York, US). A p-value <0.05 with Bonferroni correction was considered statistically significant.

## Results

**Fig 1:**
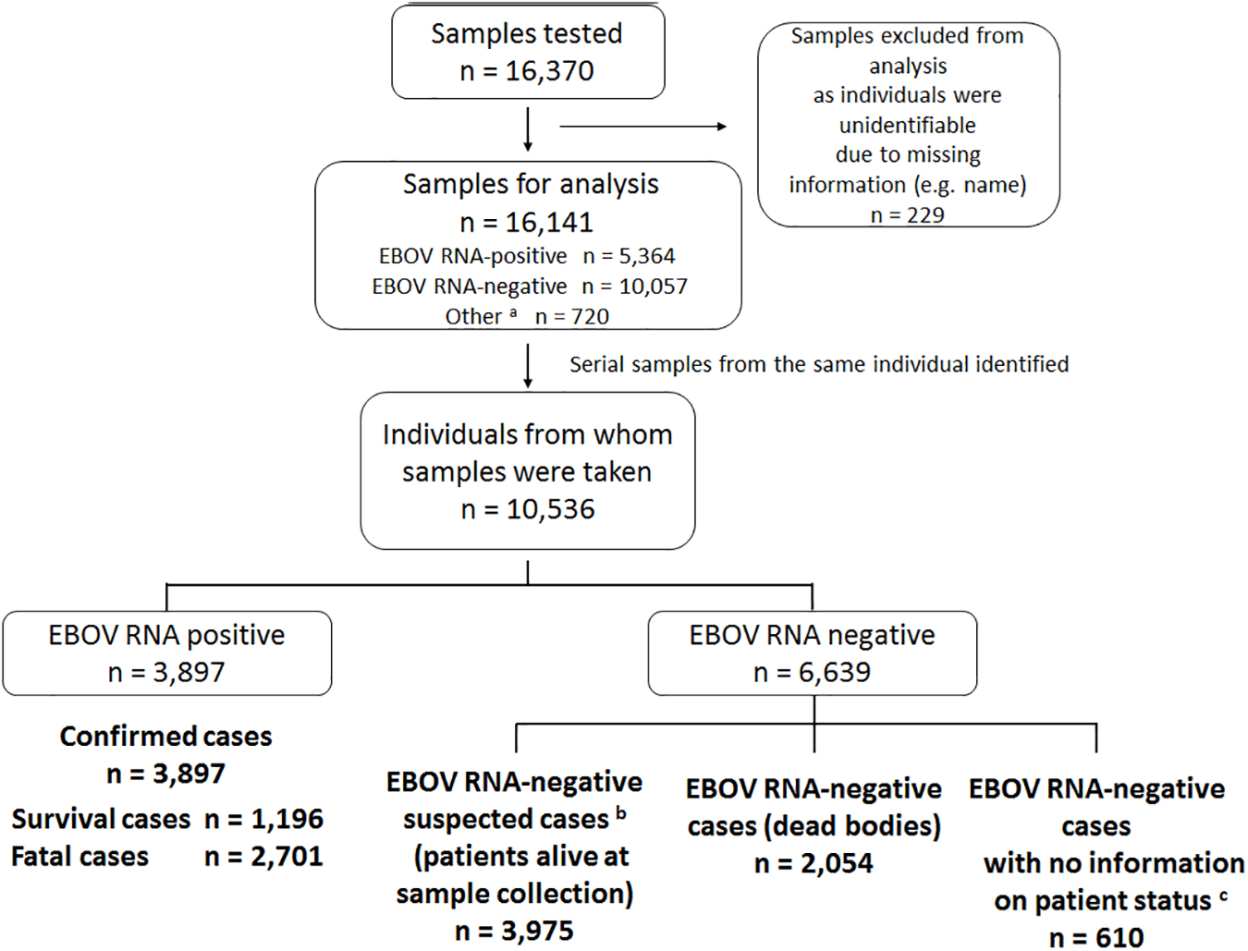
Flowchart showing processing of patient data collected by laboratories during the Ebola virus disease outbreak, Liberia, April 4, 2014–March 29, 2015. EBOV: Ebola virus. The chart shows the data processing procedure and number of cases identified. (a) Includes indeterminate results, test failures, and tests not performed due to reasons such as insufficient volume or low sample quality. (b) Comprises suspected and probable cases, because the data did not enable us to distinguish between suspected and probable cases. ^c^ Patient status refers to the patient being dead or alive at the time of sample collection.

Integrated data from the 10 laboratories contained information on 16,370 samples that were tested between April 4, 2014 and March 29, 2015. Among them, 229 samples were excluded from the analyses because individuals from whom samples had been collected could not be identified due to lack of information (such as missing names). A thorough review of the data identified a total of 10,536 individuals: 3,897 (37%) were EBOV RNA-positive and 6,639 (63%) were EBOV RNA-negative (Fig 1). A total of 2,054 persons were identified whose dead bodies tested negative for EBOV RNA, classed as EBOV RNA-negative cases (dead bodies). For 610 of the EBOV RNA-negative individuals, we had no information on whether the samples had been collected from patients who were alive at the time of sampling (EBOV RNA-negative suspected cases) or from dead bodies. We identified 3,975 EBOV RNA-negative suspected cases that probably had other acute febrile diseases such as malaria, although a differential diagnosis was not always performed. Only three of the 10 laboratories were capable of testing for malaria, and they did not always test EBOV RNA-negative samples for malaria.

We compared the profiles of EBOV RNA-negative suspected cases with those of confirmed cases, for which data were available, and found some differences (Table 1). Information on each characteristic studied varied from 49% to 80% for confirmed cases and from 57% to 91% for EBOV RNA-negative suspected cases. Approximately 52% (1,489/2,890) of confirmed cases were women or girls, whereas in EBOV RNA-negative suspected cases, the proportion was 44% (1,456/3,278; p-value <0.001). Age profiles were slightly different between confirmed and EBOV RNA-negative suspected cases (p-value <0.001). The proportion of patients who lived in the capital area was larger in confirmed cases than in EBOV RNA-negative suspected cases [73% (2,264/3,114) vs. 66% (2,405/3,620); p-value <0.001].

**Table 1:** Profiles of confirmed cases of Ebola virus disease and Ebola viral RNA-negative suspected cases, Liberia, April 4, 2014–March 29, 2015 (n = 7,872).

Characteristic	Confirmed cases ^a^		EBOV RNA-negative suspected cases		p-value ^b^
	n with characteristic/ n with available data (%)	n with available data/ total (%) ^c^	n with characteristic /n with available data (%)	n with available data/ total (%) ^c^	
Sex					
Female	1,489/2,890 (52)	2,890/3,897 (74)	1,456/3,278 (44)	3,278/3,975 (82)	<0.001
Male	1,401/2,890 (48)		1,822/3,278 (56)		
Age (years)					
<5	189/2,990 (6)	2,990/3,897 (77)	279/3,475 (8)	3,475/3,975 (87)	<0.001
6–10	221/2,990 (7)		192/3,475 (6)		
11–20	495/2,990 (17)		469/3,475 (13)		
21–30	709/2,990 (24)		826/3,475 (24)		
31–40	611/2,990 (20)		769/3,475 (22)		
41–50	432/2,990 (14)		476/3,475 (14)		
51–60	202/2,990 (7)		254/3,475 (7)		
>60	131/2,990 (4)		209/3,475 (6)		
Place of residence					
Rural areas	850/3,114 (27)	3,114/3,897 (80)	1,215/3,620 (34)	3,620/3,975 (91)	<0.001
Capital area ^d^	2,264/3,114 (73)		2,405/3,620 (66)		
Time period from symptom onset to initial testing ^e^					
Median (interquartile)	5 days (3–8)	1,914/3,897 (49)	4 days (2–8)	2,258/3,975 (57)	0.24
**Total number**	**3,897**	NA	**3,975**	NA	NA

EBOV: Ebola virus; NA: not applicable.

^a^ Confirmed by laboratory test (detection of EBOV RNA).

^b^ P-values from chi-square test or Mann–Whitney U-test as appropriate with Bonferroni correction are shown.

^c^ Showing completeness of data.

^d^ Montserrado County.

^e^ Median of the time period (days) from symptom onset to initial testing are shown.

The time period from symptom onset to initial testing for the detection of EBOV RNA was not significantly different between confirmed and EBOV RNA-negative suspected cases (p-value 0.24); approximately 75% of both types of cases were tested within seven days after symptom onset. The median time from symptom onset to initial testing was five days (interquartile range: 3–8) for confirmed cases and four days (interquartile range: 2–8) for EBOV RNA-negative suspected cases. When the clinical condition of a patient whose first sample was negative deteriorated or a physician strongly suspected EVD, another sample was collected and tested for EBOV RNA again. Among confirmed cases who were alive at the time of sample collection, 4% (140/3,136) were negative for EBOV RNA in the initial test but positive in follow-up tests. The median time from symptom onset to initial testing in such 140 patients was five days (interquartile range: 3–9).

**Fig 2:**
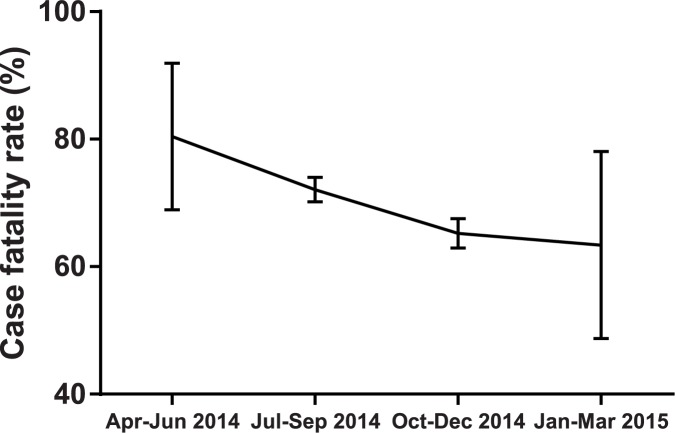
Trimonthly case fatality rate of confirmed cases of Ebola virus disease outbreak, Liberia, April 4, 2014–March 29, 2015 (n = 3,897). The p-value, calculated using the Mantel–Haenszel test for trend, was <0.001. Error bars indicate 95% confidence interval.

We also identified confirmed survival cases (n = 1,196) and confirmed fatal cases (n = 2,701), as shown in Fig 1. CFR among the confirmed cases was 69% [95% confidence interval (CI): 68–71], overall throughout the study period. CFR was higher at the beginning of the study period (80%; 95% CI: 69–92) than at the end (63%; 95% CI: 49–78) (Fig 2). This decrease in CFR was statistically significant (p-value for trend <0.001).

Comparison of patients’ profiles between confirmed survival and confirmed fatal cases showed that age (young children and the elderly) and place of residence (rural areas) were significant risk factors for fatality (Table 2).

**Table 2:** Risk factors for fatality of confirmed cases of Ebola virus disease, Liberia, April 4, 2014–March 29, 2015 (n = 3,897).

Variable	Confirmed survival cases ^a^	Confirmed fatal cases ^a^	Case fatality rate (95% CI)	Risk ratio	95% CI	p-value ^b^
Sex						
Female	551	938	63 (61–65)	Ref	NA	NA
Male	486	915	65 (63–68)	1.04	0.98–1.10	0.99
Age (years)						
<6	58	131	69 (63–76)	1.28	1.13–1.44	0.005
6–10	98	123	56 (49–62)	1.02	0.89–1.18	0.99
11–20	226	269	54 (50–59)	Ref	NA	NA
21–30	273	436	61 (58–65)	1.13	1.02–1.25	0.18
31–40	199	412	67 (64–71)	1.24	1.12–1.37	<0.001
41–50	141	291	67 (63–72)	1.24	1.12–1.38	<0.001
51–60	54	148	73 (67–79)	1.34	1.20–1.52	<0.001
>60	37	94	72 (64–79)	1.32	1.15–1.51	0.005
Place of residence						
Rural areas	239	611	72 (69–75)	1.19	1.13–1.26	<0.001
Capital area ^c^	897	1,367	60 (58–62)	Ref	NA	NA
Time period from symptom onset to initial testing ^d^						
Median (interquartile)	5 days (3–8)	5 days (3–7)	NA	0.98	0.97–1.00	0.99
**Total**	**1,196**	**2,701**	**69 (68–71)**	NA	NA	NA

CI: confidence interval; NA: not applicable; Ref: reference.

^a^ Patients who died after testing positive for EBOV RNA and dead bodies testing positive for EBOV RNA were classed as confirmed fatal cases, and patients who tested positive and then negative were considered confirmed survival cases.

^b^ P-values from chi-square test or binomial logistic regression analysis as appropriate with Bonferroni correction are shown.

^c^ Montserrado County.

^d^ Median of the time period (days) from symptom onset to initial testing and risk ratio for fatality due to one additional day in the time period are shown.

## Discussion

We identified 3,897 confirmed EVD cases and 3,975 EBOV RNA-negative suspected cases using data from laboratories during the study period between April 4, 2014 and March 29, 2015. In the WHO report, suspected (and probable) cases included patients who tested negative for EBOV RNA as well as patients who had not been sampled for the test (n = 6,561 between March 30, and March 29, 2015) [21]. Our data enabled us to reveal differences in profiles of confirmed and EBOV RNA-negative suspected cases. However, data from the laboratories was still incomplete, possibly affecting the results (Table 1). This limitation should be taken into account.

We found a larger proportion of female patients among confirmed cases than among EBOV RNA-negative suspected cases. Women and girls in Liberia have the social and cultural role of taking care of sick people in their family, and the role considered socially appropriate for women in healthcare facilities is to work as a nurse; both factors may explain the higher proportion of female patients among confirmed cases than among EBOV RNA-negative suspected cases. A high proportion of women and girls among confirmed cases in the EVD outbreak in West Africa has also been reported in other studies [22,23].

The proportion of confirmed cases living in the capital area (73%; 2,264/3,114) was higher than the proportion of EBOV RNA-negative suspected cases (66%; 2,405/3,620) in our study. This may be due to environmental factors. The capital area is a crowded environment with numerous opportunities for interaction among people and has slum areas, which increase the chances of infection, possibly leading to the massive outbreak in the capital area [24]. Although there are many reports on the epidemiology of the outbreak in the capital area [25,26] and reports from specific rural areas in Liberia [27–31], direct comparison between the areas has not been done previously. Our data allowed us to compare the epidemiological characteristics of patients in the capital area and rural areas.

We found that 4% (140/3,136) of confirmed cases who were alive at the time of sample collection were negative for EBOV RNA at the initial laboratory test but positive in follow-up tests. NATs to detect EBOV RNA can sometimes be negative for EVD cases, particularly soon after symptom onset, because of low viral titer or the presence of NAT reaction inhibitors [32,33]. Therefore, follow-up sample collection and tests, even after negative test results, should be performed for patients in whom EVD is strongly suspected clinically and/or epidemiologically.

CFR calculated in our study (69%; 2,701/3,897; between April 4, 2014 and March 29, 2015) for confirmed cases was higher than that in the WHO report for all cases (45%; 4,332/9,712; between March 30, 2014 and March 29, 2015) [21]; the WHO statistics included suspected and probable cases that had not been sampled for laboratory tests. Therefore, the cases in the WHO report probably included patients whose illness may have been due to pathogens other than EBOV, such as malaria parasites. Our data showed that CFR of confirmed cases significantly decreased throughout the study period (Fig 2). Other studies have also reported a decreasing trend in fatality among confirmed cases during the outbreak in West Africa [28,34,35]. The decrease in CFR may be attributed to improvements in medical care for EVD patients provided by various partners [15].

CFR was the lowest among confirmed cases aged between 6 and 30 years (Table 2). A high CFR among young children and the elderly was also described in other reports during the outbreak in West Africa [22,35,36]. Their insufficient immune response and/or vulnerability to severe dehydration caused by plasma leakage and diarrhea could be the reasons for the high CFR.

Living in the capital area unexpectedly turned out to be a protective factor against fatality, although the proportion of confirmed cases living in the capital area was higher than that of EBOV RNA-negative suspected cases (Table 1 and Table 2). Although environmental factors in the capital area could have increased the risk of infection, better access to healthcare (e.g., accessibility of healthcare facilities including the ETUs and the availability of medical supplies and healthcare personnel) in the capital area could have improved clinical management and thus patient outcome [15].

The limitations of this study include the fact that clustering within laboratories was not taken into account. Further, although statistical significance was calculated using a large number of samples in this study, it should be carefully interpreted for clinical meaning and significance in public health assuming independence of samples. Despite these limitations, our data yielded important findings about the EVD outbreak in Liberia, which could be of value for further epidemiological investigations and a fuller understanding of the outbreak.

## Supporting information

S1 Table Profiles of 10 EVD diagnostic laboratories in Liberia, April 4, 2014–March 29, 2015. (PDF)
