# Supplementary material for: Analysis of patient data from laboratories during the Ebola virus disease outbreak in Liberia, April 2014 to March 2015
Source: PLoS Negl Trop Dis. 2017 Jul 21;11(7):e0005804. doi: 10.1371/journal.pntd.0005804 (PMC5540615; doi:10.1371/journal.pntd.0005804)
Supplement: S1 Table — (PDF) [file pntd.0005804.s001.pdf]

S1 Table. Profiles of 10 EVD diagnostic laboratories in Liberia, April 4, 2014–March 29, 2015

Laboratory code	Location (city/town, county)	International partner [a]	Number of samples (positive, negative, other [b])	Operation period [c]
ELWA	Monrovia, Montserrado	USA	5370 (2306, 2987, 77)	Aug 2014–Mar 2015
Foya	Foya, Lofa [d]	EU	1843 (857, 968, 18)	Apr 2014–Dec 2014
Greenville	Greenville, Sinoe	USA	24 (1, 23, 0)	Dec 2014–Feb 2015
Island	Monrovia, Montserrado	USA	2108 (745, 1232, 131)	Oct 2014–Jan 2015
LIBR	Charlesville, Margibi	(USA) [e]	4980 (1483, 3298, 199)	Aug 2014–Mar 2015
Sanniquellie	Sanniquellie, Nimba	USA	150 (4, 143, 3)	Nov 2014–Feb 2015
Sinje	Sinje, Bomi	Netherlands	167 (2, 159, 6)	Jan 2015–Mar 2015
Suakoko	Suakoko, Bong	USA	1613 (274, 1316, 23)	Oct 2014–Mar 2015
Tappita	Tappita, Nimba	USA	91 (0, 90, 1)	Dec 2014–Mar 2015
Zwedru	Zwedru, Grand Gedeh	USA	24 (0, 24, 0)	Dec 2014–Feb 2015

[a] Includes governmental and non-governmental organizations.

[b] Includes indeterminate results, test failures, and tests not performed due to reasons such as insufficient volume or low sample quality.

[c] Some laboratories stayed in operation after March 2015. The period shown here indicates when samples analyzed in this study were tested in each laboratory.

[d] The mobile laboratory had been deployed in Guinea at the beginning of the outbreak and received samples from Liberia before relocation to Foya, Liberia. The present study analyzed these samples from Liberia tested in Guinea as well.

[e] The laboratory was a part of Liberian national institute and an international partner supported EVD diagnosis there.
